# “I know that I can help the person and that is priceless to me”—a qualitative study on tasks and experiences of peers in mental healthcare for refugees

**DOI:** 10.3389/fpubh.2025.1525378

**Published:** 2025-07-22

**Authors:** Lea Bogatzki, Flurina Potter, Luisa Trauner, Daniela Mier, Michael Odenwald

**Affiliations:** ^1^Department of Psychology, University of Konstanz, Konstanz, Germany; ^2^vivo international e.V., Konstanz, Germany

**Keywords:** refugees, peer support, mental health, psychotherapy, regular health care system, health care utilization

## Abstract

**Background:**

Numerous barriers prevent refugees from accessing indicated psychotherapy in the German healthcare system. Peer support emerges as a potential key element to overcome utilization barriers for refugees in the future. Since 2017, the pilot project “Coordinated Psychotherapeutic Treatment with the Involvement of Peer Support Workers” (COPEER) has been developed and implemented and is currently being evaluated. In COPEER, supervised, but autonomously acting peer support workers (PSWs) accompany and support refugees with mental disorders in the healthcare system.

**Objective:**

This study focuses on the qualitative description of the PSWs' work reality and their positive and negative work-related experiences in COPEER to assess the feasibility and relevance of their role.

**Method:**

An exploratory and qualitative approach with purposive sampling was chosen; face-to-face expert interviews were conducted using a semi-structured guide with 8 PSWs (3 women, 5 men; age *M* = 43 years). The evaluation was carried out using Mayering's Qualitative Content Analysis.

**Results:**

Five main task areas within the work reality of PSWs could be identified: organization, physical accompaniment, cultural mediation, motivation and emotional support. PSWs reported both positive and negative work-related experiences, with positive aspects being mentioned more frequently, such as high meaningfulness and a personal learning process. Negative experiences were often encountered during the initial phase of their work, e.g., problems managing the professional relationship. The mandatory continuous supervision provided by COPEER was described as helpful.

**Conclusion:**

PSWs take on important tasks that actively help to overcome central barriers to the regular health care system for refugees. In doing so, they experience both negative and positive aspects. Training and regular supervision appear to be crucial to successfully cope with work-related demands. In sum, the results show that a peer approach, as implemented in COPEER, can help to overcome barriers and enable equal access to mental health care for a particularly disadvantaged patient group.

## 1 Introduction

Refugees are considered a particularly vulnerable group in terms of the prevalence of mental disorders (1). Thus, there is a pressing need for psychotherapeutic care. However, several barriers hinder refugees' treatment in the regular health care systems. Possible solutions to overcome these barriers are coordinated care and the assistance of peer support workers (PSWs) with a comparable cultural background to the refugee.

Worldwide, there are currently over 110 million people on the run or displaced and the number of refugees has never been higher than today (2). International studies show particularly high prevalence rates for mental disorders in refugees, such as posttraumatic stress disorder (31% (3); 43% (4)) and depression (21.7% (5); 40.9% (4)).

In Germany, however, refugees' accessibility to psychotherapy provided by the regular healthcare system (defined as all healthcare providers that work under the federal social law, Code of Social Law 5 (SGB 5) and receive payments from the statutory health insurances for their services) is hindered by several barriers. There are structural barriers, such as restricted healthcare services under the Asylum-Seekers' Benefits Act, the lack of coverage of interpreter costs, administrative hurdles and a complex billing system, as well as refugees' individual and social situations, such as an insufficient knowledge of the healthcare system, differing explanation systems of symptoms and treatment expectations, stigmatization, interpersonal, institutional and structural discrimination (6, 7). In addition, on the part of the practitioners, there is a lack of training, administrative barriers and practical challenges (8), as well as, in some cases, obstructive attitudes toward therapy with refugees (9).

In general, stepped and collaborative care models are an approach to increase the accessibility to health care (7). One aspect of these models is peer support. Although there is still no uniform terminology and the concepts “peer support”, “help mentors”, “peer workers”, “peer-delivered services” or “self-help” are used differently (10), peer support crystallizes as a key element to address barriers to health services for refugees in the regular healthcare system (6). While the type of peer support may vary greatly depending on the setting and role (11), peer support in general is understood as “a system of giving and receiving help founded on key principles of respect, shared responsibility and mutual agreement of what is helpful” (12). A distinction can be made between two forms of peer support: peer-developed peer support (non-hierarchical, informal self-help) and the employment of peer staff in traditional mental health programs (10). The definition of peers varies and can differ between settings. In mental health care, peer support on an individual or group level is defined as support for people with mental health crises from another person who has lived experiences of mental health issues (11). More broadly defined, peer support is understood as support from people who share similar backgrounds, long-term conditions, or health experiences and who come together at a group or individual level to support each other (13). Peer support in working with refugees is also interpreted in various ways. According to the nationwide working group of psychosocial centers for refugees and victims of torture in Germany, the English term “peer” is understood as “equal” and defines peer counseling as support provided by people who have experienced similar or identical limiting life circumstances (14). The focus of this paper is on employed PSWs who share a common cultural background and/or an integration experience in Germany with their refugee patients.

In addition to the broad definitions and the variability of use of peers in the healthcare sector, there is great heterogeneity regarding the areas of responsibility carried out by the peers. In the general mental health care, e.g., the center for Substance Abuse Treatment's Recovery Community Support Program, four types of recovery support services carried out by peer recovery support specialists were identified: Emotional support (caring, empathy), informational support (provision of health information), instrumental support (e.g., assistance with unpleasant or stressful tasks) and companionship (e.g., helping people to feel connected) (15). The areas of peer work with refugees range from unspecific, undefined tasks such as offering space for thoughts and feelings and undertaking joint activities (16) to very specifically described peer-based interventions (such as self-help programs, including dealing with stress, relaxation, vulnerability/resilience factors, conflicts and cultural misunderstandings (17)) and vary depending on the project and target group. Thus, more research is needed to gain a more precise understanding of the responsibilities and tasks of PSWs working with refugees.

Regarding the effectiveness of peers in mental health care, there is clear evidence that peer support interventions have positive effects, e.g., self-management and peer-navigator interventions, especially in finding one's way around in the healthcare system (18). Other positive aspects show an increase in quality of life, interpersonal relationships and in supporting a more active bond in treatment (19). Creating a fast and deep build-up of trust with patients and acting as role models can be assumed to be mechanisms of action (20).

Peer support was also found to have positive effects for mentally distressed refugees (6). In the case of refugees, employed PSWs may not share a “crisis experience“ (by former diagnoses of mental disorders), but rather share a common cultural background and/or an “integration experience“ (that was already successful in the case of the PSWs). As with non-refugee patients, systematic reviews show that peer support is associated with numerous positive effects such as building new relationships, getting access to resources, receiving emotional support, talking through problems, coping with difficulties and general psychological support (6, 21). In addition, one study with community health workers found a higher patient activation, more use of medical appointments and a reduction in missed appointments (22).

Providing peer support, however, can be a source of stress to the individuals who provide it (23). Until now, research has paid limited attention to this aspect. Single studies reported that the caregivers' own emotional involvement caused stress and problems managing professional boundaries (24). Others described protective factors, such as personal growth (6, 25), training, access to self-care and supervision (24). Studies on peer support in the refugee population also showed positive effects on PSWs' personal development: through their work, their cultural understanding, empathy, interpersonal skills and problem-solving capacities increased (6, 21). Since peers are often former refugees themselves, they serve as role models for successful integration and can thus promote hope for their mentees. Peers often reported feeling empowered by sharing their experiences and serving as positive role models (16, 21).

Overall, it should be noted that there are only a few studies that explicitly deal with the benefits and burdens of peer work. While existing studies mentioned some negative experiences, more positive experiences were reported. Further research is needed to investigate PSWs' negative and positive experiences.

In a pilot project carried out by the University of Konstanz in close cooperation with the non-profit non-governmental organization vivo international e.V. (www.vivo.org), a new care model has been evaluated at a local level since 2017: Coordinated Psychotherapeutic Treatment with the Involvement of Peer Support Workers (COPEER; German: Koordinierte psychotherapeutische Behandlung unter Einbezug von Gesundheitspat:innen; KOBEG). The project implements several innovative concepts, including coordinated care and the coordinated use of trained PSWs, to make services of the regular healthcare system available to asylum seekers and refugees and to support service providers to offer treatment to them, e.g., by covering the costs of interpreters for the therapy sessions and by offering intervision.

Refugees with mental health problems are screened and diagnosed in a coordination center, their needs are assessed and they are referred to the appropriate service providers of the local regular healthcare system. Employed PSWs are bilingual, well-integrated and mentally stable lay people from the refugees' countries of origin. They are assigned to the refugee patients and accompany and support them with continuous supervision on their way into the regular healthcare system. PSWs share the cultural background, the history of flight or migration and the experience of integrating into German society PSWs share with the patient the cultural background, the history of flight or migration, and the experience of integrating into German society. Thus, the PSWs in COPEER are not defined as peers by former diagnoses of mental disorders, but by a common cultural background to the refugee patients. Preparing them for their work, PSWs receive a comprehensive training (e.g., on the German healthcare system, mental disorders, especially PTSD, self-care and professional relationship management, dealing with crises, interpreting in psychotherapy). PSWs are paid for their work and are obliged to take part in regular supervision sessions. The conceptual distinction between PSWs and interpreters is particularly important in the project: the latter are also financed and trained by the project, but are only present during therapy sessions and otherwise have no contact with the patients. The feasibility of COPEER to integrate refugees with mental disorders into the German regular psychotherapy system has currently been reported (26) and a mono-centric pilot RCT to prove its effectiveness is running (NCT04832035; clinicaltrials.gov/; April 5, 2021).

One of the central aspects concerning the evaluation of COPEER is whether its peer concept is feasible and relevant, i.e., whether PSWs take on relevant tasks for the integration of refugee patients into the regular healthcare system.

The aims of this study are (A) to describe the actual tasks which PSWs carry out in COPEER, and (B) to investigate PSWs' negative (e.g., burden, stress) and positive experiences (e.g., personal gains) associated with their work.

## 2 Methods

### 2.1 Selection of methods and study design

An explorative and qualitative design was chosen, as this research question has not yet been studied in a similar model of peer-supported care for refugees. It was assumed that interviews would provide access to the subjective views of the PSWs as an expert group. The study was thus designed and evaluated based on subjective epistemology (27). The selected methodological approach included descriptive (task areas) and evaluative (negative and positive experiences) aspects of the work of PSWs.

A semi-structured interview guide was designed according to Helfferich ((28); see Supplement S1). The interview guide included eight main areas, each with open key questions, as well as specific additional questions. In the interview guide, we also defined basic rules for the interview (e.g., narrative prompts are formulated as an introduction, the question blocks should be worked through in a flexible order, each key question should be discussed within a block, whereby the key question is only asked if not mentioned by the interviewee themselves, as well as information on dealing with follow-up questions, pauses or avoidance) and recommendations on reactions to potentially difficult situations. Moreover, an interview protocol sheet was completed by the interviewer immediately after the interview to record special incidents (e.g., breaks), the atmosphere during the interview and additional information on the setting. The interview guide was piloted in advance by project staff, i.e., a “trial interview” was conducted by the project coordinator and interviewer of the study with another psychologist project staff member.

### 2.2 Data collection

A purposive sampling strategy was chosen to reach experienced PSWs of the COPEER approach. PSWs who had been involved in the project for at least 3 years were invited by telephone to take part in individual face-to-face expert interviews. Before the interview started, extensive information was provided orally about the purpose of the study, the voluntary participation, further processing of the data and anonymization. All invited PSWs gave their written informed consent. All interviews were conducted between April and June 2022, interviewees received 25 Euros/h. The interviews were conducted face-to-face in the project office and recorded using a voice recorder. The interviews lasted on average for 71.75 min (49–90 min).

All interviews were conducted by a female psychologist and doctoral student (age 28, born and raised in Germany, Caucasian) who has held the position of project coordinator for several years. Therefore, the interviewer herself had her own views and thoughts about the project and preliminary considerations about the roles and tasks the PSWs might have. The interviewer and the PSWs had also been in contact for several years before the survey due to their close collaboration. Due to her involvement with the project and previous professional contact with all PSWs, the interviewer brought prior knowledge, assumptions, and perspectives into the data collection process. This familiarity may have influenced not only the interview dynamics but also the interpretation of the material. To critically reflect on and mitigate these influences, several strategies were implemented. The research team discussed the potential for interviewer bias and social desirability in detail. A structured, theory-based interview guide (according to Helfferich) was developed to support consistency and reduce leading questions that would guide or direct the respondent toward a certain type of answer. Moreover, the interviewer and a psychologist conducted a trial interview and training session using the interview guide to sensitize the researcher to her own biases and communication style. The interviewer's close professional relationship with the participants was recognized as having both positive and negative effects—enhancing trust and openness on one hand, considered a strength in qualitative research due to the deeper understanding of the research context, but also potentially encouraging confirmation bias, social desirable answers or hesitation to disclose negative feedback on the other. In order to address these challenges in the best possible way, detailed explanations were provided regarding informed consent, confidentiality safeguards, and the study's objectives.

### 2.3 Study sample

We selected a purposeful sample by inviting the eight individuals who had worked the longest on the project to participate. The sample included three women and five men, with an average age of 43 years (range: 25–61 years). One PSW was born in Germany, the other countries of birth were Afghanistan (1), Syria (2), Tunisia (1) and Iran (3). Their mother tongues were German, Arabic, Farsi and Dari. The seven PSWs who were not born in Germany had been living in Germany for on average 11 years (range: 5–31 years). All PSWs had already supported at least 5 refugee patients (mean = 9.8, range: 5–15) and were working with at least one patient at the time of the interview (mean = 2.6, range: 1–5). All PSWs also worked as interpreters as part of the COPEER project. For each patient, they were only active in one role at a time (PSW or interpreter). The study focuses explicitly on their role as PSWs within the project.

### 2.4 Qualitative analysis

The audio files were transcribed and anonymized using standard orthographic transcription by two psychology interns and one psychology student. The transcription was carried out using otranscribe.com (Bentley, O.D.), it was transcribed phonetically, whereby neither the grammar nor the dialect or accent of the PSWs was changed. For better readability, all anchor examples (example quotes that describe a main code category) in the code system and all quotations in the results section were also adapted to standard German and then translated into English (see Supplement S3). There was no back-translation of the transcripts. All transcriptions were proofread and checked for mistakes by the two internship psychologists and the psychology student and were listened to again by another person.

The data was analyzed using qualitative content analysis according to Mayring (29). This method enables complex information to be extracted from textual data and interpreted, whereby the analysis is carried out in eight steps: Preparation and definition of the analysis objective (clear definition of the research questions), material collection (conducting the interviews), content indexing (texts are indexed in terms of content by marking relevant text sections and recording initial content-related connections (30)), category formation (deductive-inductive categories were formed on the basis of the text materials to create a systematic order of the content (30)), rule formation and evaluation (defining the rules to apply categories, interpreting data), interpretation and reporting (presenting the results). The practical data analysis was carried out by the psychologist/doctoral student conducting the interviews and a psychology student with the help of MAXQDA (2022), a software for qualitative and mixed-methods research (https://www.maxqda.com/de).

The categories were formed deductively–inductively. This means that the main categories were initially derived from the interview guide and later supplemented inductively when analyzing the text material. In addition to the main code “task areas” and the codes “positive experience” and “negative experience”, seven other main codes from the text were analyzed and subtracted. The full code set containing all main codes developed in this study is available online (see Supplement S2). The two evaluative main codes “positive experience” and “negative experience” were only assigned in combination with another main code. The analyzed main code “task areas” with its subcodes is reported descriptively for the first research question (task areas of the PSWs). For the evaluation of the second research question (positive and negative experiences), the number of text segments (of all main codes) overlapping with the evaluative codes was analyzed. We reported the two most common code overlaps within the entire code system for the analysis of positive and negative experiences. Reflexivity was applied during data analysis. The coding process and the systematic development of the category framework were carried out collaboratively by the interviewer/doctoral student and a psychology student, accompanied by iterative team discussions to cross-check interpretations and minimize unilateral bias. The coding system was repeatedly discussed and reviewed in expert panel meetings involving four participants. These steps aimed to ensure transparency, intersubjective comprehensibility and critical distance.

### 2.5 Quality criteria

Typically used quality criteria for quantitative data cannot be directly applied to qualitative research (31) but should be reformulated for qualitative research (32). The material quality can be classified as high, as the interviews were conducted face-to-face with experts. The transcription quality can be rated at a good level, since completeness, accuracy, consistency, freedom of interpretation and comprehensibility were taken into account (33). In addition, the validity of the transcriptions was ensured by cross-checking with another person. To ensure intersubjective comprehensibility, the code system was repeatedly discussed among a group of experts (a clinical psychologist, two psychologists, psychology student); rule-guidedness was ensured by the *a priori* definition of the research questions and the study design. Theoretical guidance was partially possible due to the links to other projects and some similar studies (18, 20), as well as theoretical guidance through acquired knowledge and years of work in the project. At the same time, this is an exploratory survey, as the interviewed professional group does not yet formally exist in the regular health care system. Intracoder reliability was not determined. In order to specify the code system and to obtain more concrete code definitions, one interview was compared using consensual coding. After the intercoder discussion, a very good intercoder agreement of Cohen's Kappa of 0.92 was obtained for the analyzed interviews (consensual coding and calculation were carried out via MAXQDA). The generalizability of the results can be classified as low; the present study is primarily concerned with concrete and tangible insights of a specific small target group. The qualitative criterion of transparency was achieved through the detailed documentation of the entire evaluation process. The COREQ checklist was used for this purpose (see Supplement S4).

## 3 Results

With the help of structured qualitative content analysis, the following 10 main codes were identified: “task areas”, “negative experience”, “positive experience”, “factor PSW”, “factor patient”, “working with coordination office”, “working with healthcare system”, “value for patients”, “coping strategies” and “future” (for a description of the code set see Supplement S2). The most frequent overlapping main codes with “negative experience” were “factor PSW” and “factor patient”. The most frequent overlapping main codes for “positive experience” were “factor PSW” and “working with coordination office” (definition see below and Supplement S2). The results concerning the other main codes will be reported elsewhere.

### 3.1 Task areas

From the main code “task areas”, 5 subcodes could be inductively derived: Organization (24.7% of the total amount of mentions), accompaniment (21.9%), cultural mediator role (24.2%), motivational tasks (19.1%) and emotional support (10.1.%; see Table 1). In the following, these subcodes will be described and analyzed in more detail.

**Table 1:** Table of main code “task area”.

**Main-code**	**Sub-codes**	**Sub-sub-codes**
Task area	Organization	Making appointments
		Reminders to keep appointments
	Cultural mediator role	Cultural mediator role in therapy context
		Cultural mediator role in bureaucracy context
	Accompaniment	Accompanying in regular healthcare
		Accompanying patients to public authorities
		Appointments with refugees
	Motivation	Motivation for therapy
		Facilitating hope and/or promoting independence
	Emotional support	Emotional support of the patients

#### 3.1.1. Organization

Organizational tasks were described by all eight PSWs. The organizational work was primarily described with the thematic main focuses (subcodes) “making appointments” and “reminders to keep appointments”.

##### 3.1.1.1 Making appointments

Organizing appointments (described by all eight PSWs) involved arranging and coordinating appointments with psychotherapists, general practitioners, medical specialists and social workers. Organizing appointments was described as “*[...] time-consuming, you have to call, make appointments, sometimes it doesn't work right away and you have to ask again…” (PSW1)*.

##### 3.1.1.2. Reminders to keep appointments

Another focus was on the organizational work of reminding the patient of appointments (e.g., by sending a reminder message in advance or calling the day before, *n* = 6). Reliably keeping agreed-upon appointments was often described as a major challenge. PSWs' responses show that their role here is to build a bridge between the healthcare system and the refugee, e.g.: “*So I would often call them a week, a day ahead or the same day of the appointment and tell them that they have an appointment today and they need to be there” (PSW5)*.

#### 3.1.2 Accompaniment

The function of accompaniment can be described by the subcodes “accompaniment to regular healthcare”, “accompaniment to public authorities”, and “appointments with PSW”.

##### 3.1.2.1 Accompanying in regular healthcare

Six PSWs explicitly described having accompanied their patients regularly to appointments in the regular healthcare system (therapists, psychiatrists, or general practitioners), especially during the initial stages of treatment. The support provided by the PSWs ranged from the offer of singular to regular pick-ups and accompaniments, e.g.: “*Or [they] need to be picked up from home or where they are at that moment because they forgot. So, that's where it starts. Before therapy, you pick them up. You go with them to the therapist...” (PSW4)*.

##### 3.1.2.2 Accompanying patients to public authorities

Four PSWs stated that they had also accompanied their patients to public authorities. In this context lawyers (*n* = 3), district offices (“Landratsamt”, *n* = 2), immigration authorities and job centers, health authorities, schools and chambers of commerce were named (each by one PSW), e.g.”*[…] You may also have to go to the authorities if the patient is not insured. You have to go to the district office or the responsible authority that gives him this authorization. I've often been to the district office […] or the health authority” (PSW4)*.

##### 3.1.2.3 Appointments with PSW

Seven PSWs mentioned own appointments with patients as part of their work. They described visits at the patients' home, with the patients' families, or stabilizing positive activities in acute stress situations, e.g.: “*I said, if you're feeling bad, why don't you call me? Then we'll go out together…” (PSW8)*.

#### 3.1.3 Cultural mediator role

All PSWs described a “cultural mediator role” as part of their work. A distinction can be made between a “cultural mediator role in the therapy context” and a “cultural mediator role regarding bureaucracy and administrative processes”.

##### 3.1.3.1 Cultural mediator role in therapy context

All PSWs described an explicit transfer of specific cultural “German knowledge” in relation to the therapy process and interpersonal aspects. Dealing with experienced stigmatization (self-stigmatization and stigmatization by others) was mentioned by four PSWs. The PSWs perceived the fear of mental health treatment causing external stigmatization in the target group of refugees as stronger than in the host society, so that their work was about mitigating the stigma-related avoidance of utilization: “*If I go to a psychiatrist, then I'm crazy, then I get labeled. Stigmatization is a barrier and nobody wants to be labeled like that. Through this information transfer from me as PSW [...]* [*the patients*] *get involved and want to change what happened to them” (PSW7)*. Communicating the concept of psychotherapy and explaining data protection and confidentiality also played a central role (*n* = 4), e.g.: “*We are always afraid that the past or problems will be made public at some point. I then say: This is really secure, data protection, it will never get out. They need a lot, before they really believe it” (PSW8)*. Overall, the PSWs took over a culture-mediating role regarding traditions, rituals and behaviors experienced in the host culture (*n* = 4), e.g.: “*It's mostly non-verbal. [...] this interaction and this distance that is maintained [...] because they probably know it differently from their home countries and sometimes they are disappointed [...] and then I have to intervene and say: [...] That's just the way things are here and it has nothing to do with you as a person” (PSW3)*. Interviewees also stated that this mediation of culture-specific knowledge helped “*the therapist and patient to reach a better basis and consensus in the therapy sessions in order to continue the therapy successfully” (PSW7)*. At the same time, there was also a culturally mediating role toward the therapists concerning, amongst others, cultural taboos like sexuality and gender so that diagnostics and interventions could be implemented in a sensitive way (*n* = 2). For example: “*Unfortunately, we never talked about gender at school. So, we had no sex education [...] and it's a topic that nobody wants to talk about. And when it is now open and suddenly asked, they withdraw because they don't want to talk about it and have never had this experience” (PSW1)*.

##### 3.1.3.2 Cultural mediator role in bureaucracy context

Five PSWs also described the transfer of cultural knowledge regarding official structures in Germany. First and foremost, information about the healthcare system was important: “*They didn't even know how to get a referral. ‘What do you need a referral slip for?' or ‘When do you get a referral slip?” and also that they have the right to get a health insurance card” (PSW1)*. Other central aspects of cultural mediation are bureaucratic structures and processes *(“Time consuming…” (PSW1)); “someone who teaches them patience” (PSW4)* as well as the school system, hearings and the ‘Federal Office for Migration and Refugees'.

#### 3.1.4 Motivation

All eight PSWs described a large proportion of motivational work, primarily via the subcodes of “facilitating hope and/or promoting the patient's independence”, as well as “motivation for psychotherapy”.

##### 3.1.4.1 Motivation for therapy

A large part of the motivational work involved building up motivation regarding the referral to and utilization of therapy. Seven PSWs described building motivation for therapy as part of their work. Here, culturally sensitive aspects were mentioned (see category cultural mediator role), as well as giving psychoeducation and teaching patience. In addition to the basic motivation to undergo psychotherapy, another part of the work was to motivate patients to attend, continue treatment and not drop out: “… *they come [to the] first two or three appointments maybe.... they don't need so much motivation [from outside], but after that they need it because they thought the result would come very quickly. So, if after three or two appointments they feel that there hasn't been much development yet, they need even more motivation. ‘It takes a long time', or something like that. Or some patients don't feel like coming to an appointment or have a lot to do. I always have to motivate them and explain that it always helps, but that it takes a lot of patience” (PSW2)*.

##### 3.1.4.2 Facilitating hope and/or promoting independence

This aspect (*n* = 4) often involved conveying hope concerning the improvement of psychological suffering and functional impairments as well as general living conditions and the increasing promotion of the patient's independence and motivation for independence in the course of therapy; e.g.: “*I try to make the patients more independent. I say, okay, you do it yourself. You can do it” (PSW8)*. Using themselves as role models, PSWs conveyed hope through describing their successful integration after a difficult post-migration phase: “*When I think [back] to myself, many [refugees/new arrivals] are depressed, hopeless and don't know what the future holds. Whether this time will ever be over or not. And I tell them many of my experiences and how it was for me [to arrive] in Germany...” (PSW1)*.

#### 3.1.5 Emotional support

Seven PSWs reported emotional support of the patients as an important task. This task included building a good personal relationship, becoming a person of trust for the refugees and thereby creating support and security. At the same time, they described that active contact, listening and engaging in exchange decreased the patients' feeling of loneliness: “*When they realized that I was often available or read the messages […], I noticed: yes, they don't feel [so] alone [anymore]” (PSW6)*. Four PSWs described flexible availability and commitment when patients were stressed, either accomplished by phone or in direct contact (see category “accompaniment”, subcode “meet-ups”).

### 3.2 Positive and negative experiences

The number of coded text segments with the evaluative code “negative experiences” (Definition: circumstances, events, contacts, etc. experienced by PSWs as subjectively negative) was 106 and the number of coded text segments with the evaluative code “positive experiences” (Definition: circumstances, events, contacts, etc. experienced by PSWs as subjectively positive) was 207.

#### 3.2.1 Negative experience (NE)

The most frequent overlaps for negative experiences were found with the main code “factor PSW” (Definition: Indications of characteristics, attitudes, behavioral patterns of PSWs which appear in the context of their work as a PSW) followed by the main code “factor patient” (Definition: Indications of characteristics, life history, behaviors and backgrounds of patients who were supported from the PSWs; see Table 2).

**Table 2:** Most commonly mentioned main codes for positive and negative experiences (PE and NE) of the PSWs.

**Main-codes (Ranking based on frequency of mentions of PE and NE)**	**Sub-codes**
**Factor PSW** 1st PE (*n* = 53) 1st NE (*n* = 30)	- Boundaries/professional relationship - Role clarifications - Strong sense of responsibility - Own biography, stressful life situation - Personal development
**Factor patient** 2nd NE (*n* = 27)	- Life stories - Symptoms - suicidal tendencies - strong negativity - suffering or mistrust - attitude of denial
**Coordination office** 2nd PE (*n* = 50)	- Supervision - Trainings - Other (atmosphere, contact)

##### 3.2.1.1 NE and “factor PSW”

NEs were mentioned 30 times in combination with the code “factor PSW”. Regarding the person of the PSWs itself, primarily the aspects of managing the boundaries of the professional relationship (*n* = 6), role clarification (*n* = 3) and a strong sense of responsibility (*n* = 2) were mentioned as negatively experienced: “*‘You know, I'm not going to do that now,' or ‘That's not my job', and when you hung up, you always thought, ‘Okay, now I've broken his or her heart, maybe I could have done something here or there' ” (PSW3);* role clarifications: “*Or they often say, for example, I need more help. It's just difficult for me when they need financial help. Unfortunately, I can't help them with that and I think they're a bit disappointed in me” (PSW8)*. In addition, memories of their own biography and a potentially difficult or stressful own life situation (*n* = 4) were described as challenging and stressful in combination with their work as a PSW: “*I have to be honest, sometimes I think, how does he do that? He must be sad. Or I always compare my personal story with the patient's story. But I did an exercise for myself. Sometimes, when I can manage it, I sit alone just before or after therapy and don't go straight home. I sit alone, sometimes I cry, sometimes I look at pictures - the pictures of my home, my parents, or something” (PSW6)*.

##### 3.2.1.2 NE and “factor patient”

NEs were mentioned 27 times in combination with the code “factor patient”. With regard to the patients, their life stories were described as particularly stressful (*n* = 5): “* […] when they tell about the army, soldiers or arrest, - these things are difficult for me” (PSW6)*. In addition, the symptoms, specifically suicidal tendencies, strong negativity and suffering or mistrust, as well as an attitude of denial, were experienced as stressful: “*First, it's sad to see people down. To hold out a hand and ask them to get up is also not very easy. Because there comes only rejection and there comes an energy, or a feeling: ‘Leave me alone, I don't believe you now, you're only here for your own benefit' ” (PSW3)*.

#### 3.2.2 Positive experience (PE)

The most frequent overlaps with positive experience were found with the main code “factor PSW”, followed by the main code “working with the coordination center” (Definition: Information on the experiences of the PSWs in cooperation with the coordination office; see Table 2).

##### 3.2.2.1 PE and the “factor PSW”

PEs were mentioned in 53 segments in combination with the code “factor PSW”. In terms of the PSWs' personalities, the personal development throughout the project and work (e.g., social skills, increase in specialist knowledge, broadening of perspective) was experienced by all eight PSWs as positive: “*Learning psychological things, it's so instructive for me” (PSW3)*. The fact that they were able to rely on their own personal experiences and life stories also proved enriching. A successful clarification of boundaries and the associated learning of more self-care as well as enjoying the responsible role were marked positively, e.g., “*This is very nice for me personally, because I generally like to take on such a role in life. Also toward my family and my colleagues. I just want everyone to look ahead and move forward*” *(PSW3)*; associated learning of keeping boundaries, e.g., “*So for me [it] was very good, a very good experience. I've been here for so many years now and I already realize who needs what or how far I'm allowed to go in, I can judge the distance better [...] I realize this better now than at the beginning and [with] the role I have [...] I get along better than at the beginning” (PSW1)*. Furthermore, self-efficacy and motivation were also experienced positively, “*because I know that I can help the person, and that is priceless to me” (PSW8)*.

##### 3.2.2.2 PE and work with the coordination office

PEs were mentioned 50 times in combination with the code “working with coordination office”. First and foremost, all eight PSWs mentioned the trainings within the project and the acquired specialist knowledge as helpful: “*These training courses are extremely important” (PSW1*) and helped the PSWs to prepare for their work, e.g., “*[…] very, very helpful training courses. Yes, that's the feeling that you're not thrown in at the deep end […]” (PSW5)*. In addition, the regular supervision through the exchange with other PSWs was described as enriching (*n* = 7), as were the atmosphere, mutual support, social interaction and an understanding, open handling within the team; e.g., “*Supervision is absolutely the best, whether as a translator or as a PSW. […] I love it. I will always participate when I can […]” (PSW8)*, “*Good. Communicative, helpful. Because those who have less experience […] get advice or tips from others on how to deal with the situation” (PSW7)*.

## 4 Discussion

### 4.1 Summary of results

The in-depth analysis of the eight qualitative interviews shows that the PSWs take over important tasks supporting refugee patients utilizing services of the regular health care system. Overall, their tasks comprise three areas, i.e., practical assistance to arrange and keep appointments (see codes “organization” and “accompaniment”), facilitation of cultural exchange between clients and service providers (“cultural mediator role”), and tasks supporting the therapeutic process, building up treatment motivation and provision of emotional support (codes “motivational tasks” and “emotional support”).

PSWs have both negative and positive experiences, with positive experiences being mentioned more frequently. Negative experiences were primarily associated with personal challenges in terms of management of their own boundaries, responsibility and role clarity, as well as with the characteristics of the patients (life history and severity of symptoms) and occurred mainly in the beginning of their work as PSWs. Positive experiences were evident in relation to personal development, enjoyment of responsibility and motivation, as well as in cooperation with the coordination office.

### 4.2 Task areas

The PSWs' job includes the facilitation of a broad range of barriers to mental health service utilization.

Organizational domain: One of the main tasks of the PSWs is “organization”, including the support for regular and reliable utilization of services in the regular healthcare system. The importance of peers taking on organizational tasks has been discussed in previous studies, showing that peer support can help patients to better find their way around the regular healthcare system (18) and to avoid missing appointments (22). These results are particularly relevant, as practitioners in the regular healthcare system often complain about missed appointments with refugee patients (34). PSWs could build a central bridge to reliably bring refugees to appointments in the regular healthcare system. The aspect of “accompaniment to public authorities” seems to be closely related to the utilization of health services, i.e., the forms of assumption of health care costs by public authorities or the renewal of residence permissions. The “appointments with PSW” seem to have the quality of personal meetings, in which refugees explain their problems. This is the base for the above-mentioned organizational tasks, but also contributes to the other two task areas because here PSWs explain the German health care system, promote motivation and provide emotional support. In summary, the organizational domain covers the areas of “informational support” (educational) and “instrumental support” (supporting stressful tasks, e.g., when filling out applications) conceptualized by the center for Substance Abuse and Mental Health Treatment's Administrations of Recovery (SAMHSA; (15)).

The aspect of the “cultural mediator role” can also be found in the literature on refugees, particularly in the case of psychotherapy with interpreters. Gartley and Due (35) found that interpreters do more than just translate, they seem to act as “cultural brokers” and Miller et al. (36) also assumed that language mediators clarify cultural misunderstandings and contribute to mutual understanding. Cultural mediation could play a decisive role, particularly with regard to current findings on culturally differing concepts of mental illnesses such as trauma sequelae (37). This area of responsibility addresses barriers on the refugees' and their families' part to utilize the regular healthcare system, such as an insufficient knowledge of the existing services, of medical explanation of symptoms and of adequate treatment expectations. Furthermore, PSWs can discuss and modify fears of stigmatization, interpersonal, institutional and structural discrimination (6, 7). Our results align with research, showing that PSWs informed their mentees about the healthcare system and specific aspects (e.g., confidentiality, interpersonal relationships), that they provided psychoeducation and fostered destigmatization regarding mental illness. In addition, PSWs facilitated the exchange of information between service providers and patients (see subcodes: “cultural mediator role in the therapy context” and “cultural mediator role regarding bureaucracy and administrative processes”). It can be assumed that “cultural mediation” reduces barriers for therapists and patients, contributes to more mutual understanding (36), leads to more compliance and motivation and is associated with the finding of a more active tying in treatment. Regarding these findings, it should be noted that the roles of cultural mediation and language mediation are clearly separated in the COPEER project, so that interpreters may be relieved of the cultural mediation work of the PSWs.

The third domain of the PSWs' tasks “motivation” and “emotional support” relates to the tasks “emotional support” and “companionship” propagated by the Center for Substance Abuse and Mental Health Treatment's Administration's office of recovery (SAMHSA; (15)). “Motivation” and “emotional support” are traditionally provided by highly trained mental health care providers such as psychologists, social workers or medical doctors. In the context of scarcity of human resources, the concepts of task shifting and task sharing have been introduced, for example by the WHO (38), meaning that less trained staff or trained laypersons take over defined tasks. A recent meta-analysis provided evidence for the effectiveness of non-professional task sharing in relation to different interventions, e.g., behavioral activation (39). Anvari et al. (39) highlighted the feasibility and acceptability of non-professional evidence-based interventions delivered in the context of depression, substance use, loneliness, trauma and for people with comorbid physical illnesses. Task-sharing approaches with refugees also showed positive effects, for example, in the PROSPER study, in which the World Health Organization Problem Management Plus (PM+) intervention was carried out by peer lay therapists with lived experience of seeking asylum or of migration (40).

Other studies reported that the motivational work of PSWs can lead to a more active tying in treatment (19). The “emotional support” task is closely related to the building of a trusting relationship and to the communication of hope. The PSWs serve as role models, having experienced flight/migration, having achieved integration in the host society and thereby having a strong influence on creating hope for future improvements in the refugees' lives (20).

It is evident that the tasks identified in this study are manifold and complex and that PSWs need a complex and diverse knowledge and skill base for their work. Consequently, the demand for specific training of PSWs as well as for continuous supervision and training is obvious. Therefore, a PSW training curriculum should be guided by the existing proven schemes, e.g., the comprehensive training of EXIN (experienced involvement) peers. The concept for a curriculum for the training of EXIN peers was developed internationally and includes defined theory lessons (12 units, 22 h) and practical internships of 120 h (41). The training content includes some of the following topics, e.g., empowerment, participation, trialogue, recovery, research, assessment, accompaniment and support, crisis intervention (42). These training modules overlap with some aspects that PSWs also adopt in their work with refugees. Other recommendations on training (quality of training and content of training), certifications and implementation of PSWs in the healthcare sector are available from the Substance Abuse and Mental Health Services Administration (SAMHSA) Office of Recovery, U.S. Department of Health and Human Services (43).

The training of PSWs in COPEER comprises 15 h, plus three additional hours for language mediation training (as many PSWs also work as interpreters in the project). For the further development of the peer concept in Germany, legal foundations, organizational basics of recruitment and training for PSW in the mental healthcare system with refugees are necessary.

In summary, PSWs reported taking on central tasks of different complexity and in different domains of barriers to healthcare utilization that have already been reported in the literature (6). The initial evaluation results from the COPEER model show that typical barriers to service utilization are successfully addressed, e.g., a low drop-out rate of refugee patients and a high commitment of psychotherapists to treat them with project support (26), which is certainly also the result of the PSWs' work. Our results also confirm that PSWs need a broad knowledge and skill base achieved with a comprehensive training curriculum and that they require continuous supervision.

### 4.3 Negative and positive experiences

PSWs reported positive and negative experiences in their work, whereby the fact that positive experiences are mentioned more frequently might reflect that they outbalance the negative experiences in the PSWs' work.

In relation to their own person, the following aspects were mentioned as negative work-related experiences: the management of the professional relationship and its limits, role clarification, a too strong sense of responsibility, the provocation of stressful memories of one's own life, as well as emotional overload due to the PSWs' own current situation. Previous studies also reported the setting of boundaries and role ambiguity as challenging for PSWs (24). Negative experiences in relation to the patients were particularly evident regarding the burden of getting to know their life stories and severe symptoms, especially suicidal tendencies. Emotional involvement is evident and has been reported in other studies with PSWs (24).

In the analysis and consideration of the positive experiences, it is to be noted that some aspects initially described as negative (especially in relation to the PSWs themselves, “factor PSW”) are subject to change and are later reported as positive. For example, some PSWs described it as positive to have learned to be able to rely more on their own personal experiences and life stories. Moreover, they also experience a positive learning experience in relation to improved management of their own boundaries and self-care, as well as to higher enjoyment of the PSWs' responsibility. Here the PSWs personal growth is evident: With ongoing training, supervision and acquired skills, burdens and difficulties regarding responsibilities, boundaries, role clarity and one's own story seem to become manageable. This leads to initial burdens to be perceived as very positive and empowering throughout their work. This learning and associated “personal growth” is evident in various areas (e.g., social skills, increase in specialist knowledge, broadening of perspective) and has already been found in other studies with PSWs (6, 21). The regular supervision, peer-to-peer exchange, the training and further education provided by the project are also seen as positive. In line with other studies, it can be assumed that training and supervision have a protective effect on the stress of PSWs (24).

In summary, it can be stated that PSWs have both negative and positive experiences, whereby regular training and supervision appear to be important for a learning process and personal growth (shifting from negatively challenging experiences of managing boundaries and clarifying roles to self-determined actions and enjoyment of responsibility).

### 4.4 Limitations

Some methodological limitations need to be discussed. On the basis of subjective epistemology, it can be assumed that the interviews provide insights into the subjective perception of the PSW and do not reflect objective reality. What counts here is the “subjective truth” and the principle of “benevolent interpretation” or “meaningfulness assumption” (28). However, it should be noted that perception can be influenced by various effects such as social desirability, memory bias and selective perception.

A recent systematic review found that data saturation in qualitative studies is often achieved with relatively small sample sizes, typically ranging from 9 to 17 participants for interviews and 4–8 participants for focus group discussions (44). Other studies suggest that a sample size of twelve may be sufficient to reach content saturation, while basic meta-themes can already be identified with as few as six interviews (45). Based on these findings, it can be assumed that a sample of eight PSWs is sufficient to identify meta-themes. As Malterud et al. (46) argue, according to the concept of “information power”, the more relevant and information-rich a sample is in relation to the research question, the fewer participants are needed to produce meaningful data. In the present study, the expert sample was narrowly defined and highly specific. It was selected with the explicit aim of obtaining focused expert insights. Thus, only PSWs who had been involved in the project for several years were selected and invited to participate. Therefore, a high level of information power can be assumed. It can thus be assumed that data saturation was achieved as far as possible under the given circumstances. However, the results should be interpreted with caution, as our study examined a sample below the recommended sample size of 9–17 (44) for interview studies, and the findings require replication before they can be generalized more broadly.

The semi-structured interview guide (according to Helfferich, (28)) asked directly about some of the PSWs' areas of responsibility, which may have led PSWs' answers in one direction. A cross-validation of the findings through the perspectives of involved patients or therapists is lacking and would be essential for further evaluation. It should also be noted that the sample of PSWs overlaps with the interpreters in the project. Although a clear distinction between PSW and interpreter roles was sought, this dual engagement may have influenced role perception and reporting. Furthermore, the interviews were conducted by the project coordinator herself, who has been actively working with all the PSWs for years. In this context, in particular social desirability could be suspected, which could have influenced the interviews/answers. However, we managed this potential problem to the best of our abilities, by discussing these issues openly, an extensive informed consent, confidentiality and an atmosphere of trust.

The dangers of an “interviewer bias”, a distortion of the collected and interpreted data due to the interviewer's personal expectations and prejudices were intensively discussed in the research team. In order to reduce the impact of these potential problems, we conducted a “trial interview” in advance, had expert discussion rounds and analyzed the results in a team process. Moreover, the research team continuously engaged in reflexive discussions regarding the interviewer's dual role, the power dynamics in the interview setting, and the influence of prior professional relationships on data interpretation. Positionality and potential biases were explicitly addressed during the coding process to reduce the influence of pre-existing assumptions on interpretations. These reflexive practices were understood as essential to maintaining methodological rigor and transparency and to increasing the credibility and trustworthiness of the findings. For allowing a focus on the task areas and positive and negative experiences of the PSWs, the results and discussion of the interviews do not cover the entire code system. However, to ensure completeness and transparency, all main codes and their descriptions are presented in Supplement S2.

## 5 Conclusion

This study is important for the further development and improvement of COPEER and other projects involving PSWs, as it highlights potential burdens on PSWs and offers valuable insights for developing measures in training, guidance, and support.

In an initial approach, it seems that PSWs take on central tasks that can actively help overcome refugees' barriers to utilizing services in the regular healthcare system. In doing so, they have both negative and positive experiences, whereby a learning process and the positive effects of training and supervision can be observed. Further studies are needed to evaluate the task areas and the positive and negative experiences of PSWs to achieve greater generalizability of the results. Research concerning the effectiveness of PSWs in mental health care for refugees should be conducted in a controlled trial. Additionally, examining the perspectives of refugees who received support from PSWs would provide valuable insights into the nature of the support and areas of responsibility.

Based on our findings, we recommend that PSWs find more use in the regular health care system, to provide refugees a less-discriminatory access to the human right of health. In order to include PSWs as service providers into the regular health care system, a standardized training schedule should be developed and the legal framework for their employment in the health care system needs to be set. We recommend that comprehensive and standardized trainings particularly focus on aspects of local healthcare structures, mental disorders, cultural mediation and motivational conversation management, as well as strategies for managing personal stress and setting professional boundaries. Additionally, ongoing training (to further deepen knowledge and skills acquired in practice), regular supervision and permanent support offers from a responsible and coordinating institution are needed.

## Author's note

LB is a clinical psychologist who conducted the interviews and had been collaborating with the peer support workers and coordinating the project for three years at the time of the interviews.

## Data Availability

The original contributions presented in the study are included in the article/Supplementary material, further inquiries can be directed to the corresponding author.
