# Supplementary material for: “I know that I can help the person and that is priceless to me”—a qualitative study on tasks and experiences of peers in mental healthcare for refugees
Source: Front Public Health. 2025 Jul 22;13:1525378. doi: 10.3389/fpubh.2025.1525378 (PMC12321529; doi:10.3389/fpubh.2025.1525378)
Supplement: Supplementary file 1 [file Data_Sheet_1.docx]

**Supplement 1

Expert Interview with Peer Support Workers (PSWs) of the Integration Project**
**Main Questions:** Experiences of the PSWs through and with their work in the integration project (areas of responsibility and personal experiences)

🡪 Estimated duration of the interview: 60–90 minutes

| **Category** | **Prompt for storytelling** | **Definition – Guiding Questions**  **Check: Was this mentioned?** | **Specific follow-up questions** |
| --- | --- | --- | --- |
| **K0: Introduction**  **Areas of responsibility of the Peer Support Workers (PSWs)** | "What tasks did and do you take on as a PSW?"  **Interview Rule:** If yes – ask for more details  **Note:**  Avoid overly evaluative questions! | **Tasks in the work**    **Organization**  **Motivation**  **Language mediation**  **Cultural aspects** | **Specific follow-up questions**   - *How was that for you? What was your experience?* - *What did this look like in your day-to-day life?* - *Can you explain that with an example?*   *"Did you have organizational responsibilities? How was that for you?"*  "Did you have the task of motivating people? How was that for you”  "Did you have to interpret / provide language mediation?"  "Did you mediate between cultures?" If culture is mentioned: *What does that specifically look like in your everyday work?* |
| **K1: Added value through support by the PSWs?** | "What feedback did you receive from patients about your work?" | **Patient feedback**  **Own perception** | "In what ways did the patients benefit from your work?"  "Which aspect of your work helped the patients the most, in your opinion?"  "What do you think you have achieved with and/or for the patients?" |
| **K2: Personal experience – Burden vs. Enrichment** | "How did you personally experience caring for refugee patients as a PSW?"  "Is there anything you have found or are finding burdensome in your work as a PSW?"  "What difficulties have you experienced or are experiencing in your role as a PSW?"  "Is there anything you have found or are finding enriching in your work as a PSW?" | **General experiences**  **Burdens**  **Dealing with traumatic refugee stories  Daily burden level**  **Challenges**  **Most difficult moment**  **Roles and boundaries**  **Sense of responsibility**  **Enrichment**  **Personal development** | **Open question**  **Burden**  "How do you handle the life stories of the patients?"   "Do you feel your stress level has changed?"  **Challenges**  "What was your most difficult situation?" Follow-up: *How did you deal with it / how was it resolved?*  "How did/do you experience setting boundaries with patients?" *What helps you set boundaries?*  "How did/do you experience your responsibility?"  **Enrichment**  "What was your most beautiful moment as a PSW so far?"  "What were your moments of success?"  "What has the experience as a PSW meant for your development (professionally, personally)?"  "How have your experiences as a PSW influenced your further development?" |
| **K3: Questions about the PSWs‘ personality** | "What is especially important to you in your work as a PSW?" | **Personal motivation**  **Characteristics of future PSWs** | "What is your personal motivation for working as a PSW?"  "What qualities should a PSW have, in your opinion?" "What advice would you give to future PSWs?" |
| **K4: Questions about working with the formal healthcare system** | "How did/do you experience the collaboration with the regular healthcare system in the district (contacts with therapists, doctors)?" | **Acceptance / reception** **Feedback from the system** | "What feedback did you receive from therapists/doctors about your work as a PSW?" |
| **K5: Perspective on the authorized coordination office and employer (university)** | "How did/do you experience the collaboration through the project, the coordination?"  Note to the interviewer: Use distancing method at the start as the question may be personal. | **Employer’s handling of PSWs**  **Trainings**  **Supervision**  **Wishes** | "How did/do you experience the support offers?"  "Looking back: How do you assess the trainings in preparation for the work?" *What was helpful? What wasn’t?* "Do you have suggestions for improvement?"  "How did/do you experience regular supervision/support sessions?" *Helpful? Not helpful? Wishes?*  "What would you have wished for in terms of support from the project?" |
| **K6: Conclusion – Future** | "What do you wish for the future of the project?" |  | **Open question** |
| **K7: Anything else** | "Did we forget anything you would like to mention?" |  | **Open question** |

**Interview Protocol Sheet** **(!!!TO BE FILLED OUT AFTER THE INTERVIEW!!!)**

**Interviewer:** ____________ **Date:** ___________ **Duration:** _________ min **Location/Setting:** ______________________

**Motivation for Participation:** ______________________

**Additional Information**

**Special occurrences during contact or in the interview:**

**Interview Atmosphere:**

**Keywords regarding the personal relationship:**

**Interaction during the Interview:**

**Challenging Sections:**
