# Supplementary material for: “I know that I can help the person and that is priceless to me”—a qualitative study on tasks and experiences of peers in mental healthcare for refugees
Source: Front Public Health. 2025 Jul 22;13:1525378. doi: 10.3389/fpubh.2025.1525378 (PMC12321529; doi:10.3389/fpubh.2025.1525378)
Supplement: Supplementary file 2 [file Data_Sheet_2.docx]

**Supplement 2**

**CODE SET**

The entire code set (10 main codes with definitions, coding rules and anchor examples), developed and subtracted from the interviews, is listed below.

| Code | Definition | Quote |
| --- | --- | --- |
| Task areas | Indications of activities carried out by the Peer Support Workers (PSW) as part of the project. | *“I am responsible for all the appointments I schedule here and with the doctors. I always tell the patients a few days in advance that we have an appointment, and he should remember that. He should be punctual, or I offer to pick him up.” (PSW8)* |
| Negative experience | Circumstances, events, contacts, etc. experienced by PSWs as subjectively negative.  Coding rule: May only be assigned in combination with another main code. | *“What is always burdensome for me is the intense suffering of people, no matter the cause. When I see people who are worn out early in the morning, when you see them slumped over, crying, or looking unkempt and so on. That brings me down and burdens me a lot. That’s all.” (PSW3)* |
| Positive experience | Circumstances, events, contacts, etc. experienced by PSWs as subjectively positive.  Coding rule: May only be assigned in combination with another main code. | *“But now I say, life is really beautiful. You just have to work at it a little harder and try to help others. There are many points where I say it really made me very happy. When I was working here on the project.” (PSW8)* |
| Factor PSW | Indications of characteristics, attitudes, behavioral patterns of PSWs which appear in their context of the work as a PSW. | *„But right now, I can very well imagine that I won‘t fall back into my past.” (PSW8)* |
| Factor patient | Indications of characteristics, life history, behaviors and backgrounds of patients who were supported by the PSWs. | *“It also depends on each patient and what kind of case it is. But right now it is great. The patient is a young Iranian, who was also severely traumatized, had bad experiences, and was also in psychiatry here. But slowly, his health condition is improving, and he is attending a German course.” (PSW1)* |
| Working with coordination office | Information on the experiences of the PSWs in cooperation with the projects’ coordination office. | *“It was quite good. I don’t have any problems at all. If I need anything, or if I realize that it is getting too much for me, I get in touch and then everything works out.” (PSW3)* |
| Working with healthcare system | Information on the experiences of the PSWs in cooperation with stakeholders within the healthcare system. | *“Those who understood what a PSW was found it beneficial.” (PSW7)* |
| Value for patients | Indications of experienced positive added value for patients through the work of PSWs. | *“In retrospect, I have achieved a better quality of life. Before the therapy or before my work, the patient was exposed to general and health risks and threats. Through this work, activity, and therapy, they benefited, perhaps resulting in them being 100 percent healed and thus having a better, qualitative, or relieved life. That is my opinion.” (PSW7)* |
| Coping strategies | Information on how PSWs deal with working conditions, special incidents, stress and/or difficulties which appear in the context of their work as a PSW.  Coding rule: May only be assigned in combination with another main code. | *“I try to learn something from it. I try to look on the positive side and somehow compensate for it with my help. And to make it nicer for them (the patients), that makes it nicer for me too. Most of the time I also have to tell myself that the person has unfortunately had bad luck. Sometimes things happen that cannot be undone.” (PSW3)*  *“You just have to tell yourself: yes, that’s sad and you just have to be grateful that it didn’t happen to yourself. And just hope that this person will somehow get over it.” (PSW3)* |
| Future | Statements of the PSWs regarding the future of the project. | *“What do I wish for? Well, I really wish supervision takes place at least once a month. If possible, everyone should attend…” (PSW8)* |
