# Supplementary material for: “I know that I can help the person and that is priceless to me”—a qualitative study on tasks and experiences of peers in mental healthcare for refugees
Source: Front Public Health. 2025 Jul 22;13:1525378. doi: 10.3389/fpubh.2025.1525378 (PMC12321529; doi:10.3389/fpubh.2025.1525378)
Supplement: Supplementary file 3 [file Data_Sheet_3.docx]

**Supplement 3

Quotes: Interviews Peer Support Workers (PSW)**

All quotes cited in the study are listed below in the order they appear in the text. Some of the quotations here are somewhat longer to show the "context". The final excerpts quoted in the study are highlighted in green.

| **Position**  **in Paper** | **Original** | **Standard German** | **English** |
| --- | --- | --- | --- |
| CODE  1A. Organization  SUBCODE A.1 Making appointments | *„wenn ich weiß, er braucht eine Hilfe in irgendwelche Bereich, natürlich ich werde das organisieren, Termin vereinbaren [….] ja, das ist schon zeitaufwendig, muss man anrufen, Termine vereinbaren, manchmal klappt das nicht sofort und genau, man muss nochmal nachfragen, ob es Termine gibt.“ (GP1)* | *„wenn ich weiß dass er Hilfe braucht in irgendeinem Bereich werde ich das natürlich organisieren, Termin vereinbaren [….] ja, das ist schon zeitaufwendig, man muss anrufen, Termine vereinbaren, manchmal klappt das nicht sofort und man muss nochmal nachfragen[…].“ (GP1)* | *„if I know that he needs help in any area I will of course organize it, make an appointment [....] yes, it is time-consuming, you have to call, make appointments, sometimes it doesn't work right away and you have to ask again […].” (PSW1)* |
| CODE  1A. Organization  SUBCODE A.2 Reminders to keep appointments | *„ich hab immer über die Termine weil manche sind ohne die Medikamente zum Beispiel nicht in der Lage um die Termine zu erinnern oder so was dann hab ich oft so gemacht also eine Woche vor der Termin, ein Tag vor der Termin und am gleichen Tag also dass ich der Patient angerufen haben und dann gesagt ja die haben heute einen Termin die müssen da sein oder wenn ich nicht dabei bin also.“ (GP5)* | *„ ich habe immer an die Termine erinnert, weil manche [Patient:innen], sind beispielsweiße ohne Medikamente nicht in der Lage sich an die Termine zu erinnern. Also habe ich sie oft eine Woche, ein Tag oder am gleichen Tag des Termins angerufen und ihnen gesagt, dass sie heute einen Termin haben und da sein müssen, auch wenn ich nicht dabei bin.“ (GP5)* | *"I always reminded them of the appointments because some [patients], for example, are not able to remember the appointments without medication. So I would often call them a week, a day ahead or the same day of the appointment and tell them that they have an appointment today and they need to be there, even if I'm not there." (PSW5)* |
| CODE  1B. Accompaniment  SUBCODE B.1 Accompanying in regular healthcare | *„oder müssen von zu Hause abgeholt werden, oder von wo sie auch sind, weil sie es vergessen haben. Also da geht schon los. Also vor der Therapie holt man sie ab. Man geht mit denen zum Therapeuten…“ (GP4)* | *„oder [sie] müssen von zu Hause abgeholt werden, oder wo sie eben sind, weil sie es vergessen haben. Also, da geht‘s schon los. Vor der Therapie holt man sie ab. Man geht mit denen zum Therapeuten…“ (GP4)* | *"Or [they] need to be picked up from home or where they are at that moment because they forgot. So, that's where it starts. Before therapy, you pick them up. You go with them to the therapist..." (PSW4)* |
| CODE  1B. Accompaniment  SUBCODE B.2 Accompanying patients to public authorities | *"Dann eben dieser ganze Verwaltungsaufwand, ja? Mit den Patienten zur Therapie und... gerade so kann es auch sein, dass man dann Behördengänge machen muss, wenn der Patient nicht versichert ist. Dass man doch Landratsamt geht oder zum zuständigen, zur zuständigen Behörde, die ihm halt eben diese Bewilligung gibt. Hmm ich war schon oft bei Landratsamt in Radolfzell, beim Gesundheitsamt." (GP4)* | *"Dann eben dieser ganze Verwaltungsaufwand, ja? Mit den Patienten zur Therapie und ... es kann auch sein, dass man dann Behördengänge machen muss, wenn der Patient nicht versichert ist. Dass man zum Landratsamt geht oder zur zuständigen Behörde, die ihm diese Bewilligung gibt. Ich war schon oft beim Landratsamt (in Stadt), oder beim Gesundheitsamt." (GP4)* | *“Then all this administrative work, right? Taking patients to therapy and ... You may also have to go to the authorities if the patient is not insured. You have to go to the district administration office or the relevant authority that gives them the permit. I've often been to the district office (in the city) or the health authority." (GP4)* |
| CODE  1B. Accompaniment  SUBCODE B.3 Appointments with PSW | *„wo ich der gesagt habe, wenn es dir schlecht geht, dann ruf doch mich an. Dann gehen wir zusammen raus. […] Der hat auch gesagt, ok, dann gehen wir jetzt raus.“ (GP8)* | *„als ich gesagt habe, wenn es dir schlecht geht, dann ruf doch mich an. Dann gehen wir zusammen raus […] Dann hat er auch gesagt, ok, dann gehen wir jetzt raus.“ (GP8)* | *"When I said, if you're feeling bad, why don't you call me? Then we'll go out together [...] He then said: ok, then we'll go out now." (PSW8)* |
| CODE  1C. Cultural mediator role  SUBCODE  C.1. Cultural mediator role in therapy context | *„Wenn ich dann zu einem Psychiater gehe, dann bin ich dann verrückt ich würde abgestempelt ich bin verrückt. Stigmatisierung ist dann eine Barriere und keine will das so abgestempelt werden und dadurch durch diese Informationsvermittlung von mir als GP oder vielleicht darüber hinaus sie lassen sich darauf ein und möchten ändern was ihnen passiert ist.“ (GP7)* | *„Wenn ich zu einem Psychiater gehe, dann bin ich verrückt, dann werde ich abgestempelt. Stigmatisierung ist eine Barriere und keiner will so abgestempelt werden. Durch diese Informationsvermittlung von mir als GP [...] lassen sich [die Patient:innen] darauf ein und möchten ändern, was ihnen passiert ist.“ (GP7)* | *"If I go to a psychiatrist, then I'm crazy, then I get labeled. Stigmatization is a barrier and nobody wants to be labeled like that. Through this information transfer from me as PSW […] [the patients] get involved and want to change what happened to them." (PSW7)* |
| CODE  1C. Cultural mediator role  SUBCODE  C.1. Cultural mediator role in therapy context | *„Wir haben immer Ängste, dass sie irgendwie ihre Vergangenheit oder ihre Probleme irgendwann veröffentlicht. Und wo man auch da sagt, hier ist wirklich gesichert. Datenschutz. Das kommt niemals raus. Sie brauchen viel bis sie wirklich dran glauben.“ (GP8)* | *„Wir haben immer Ängste, dass die Vergangenheit oder Probleme irgendwann veröffentlicht werden. Ich sag dann: hier ist das wirklich gesichert, Datenschutz, das kommt niemals raus. Sie brauchen viel, bis sie wirklich dran glauben.“ (GP8)* | *“We are always afraid that the past or problems will be made public at some point. I then say: this is really secure, data protection, it will never get out. They need a lot before they really believe it.” (PSW 8)* |
| CODE 1C. Cultural mediator role  SUBCODE  C.1. Cultural mediator role in therapy context | *„Es ist meistens nonverbal. Ja. Es ist…dieser Umgang und diese Distanz, wo eingehalten wird, natürlicherweise und manche Menschen denken, weil sie dann zum Beispiel in einem Rahmen, also dass dann in diesen Rahmen soll es jetzt anders sein, weil sie wahrscheinlich das anders kennen aus ihren Heimatländern. Und manchmal sind sie halt bisschen so enttäuscht […] So ist halt der Umgang hier und das hat mit deiner Person nichts zu tun." (GP3)* | *"Es ist meistens nonverbal. […] dieser Umgang und diese Distanz, die eingehalten wird […] weil sie das wahrscheinlich anders kennen aus ihren Heimatländern und manchmal sind sie dann enttäuscht […] und da muss ich dann eingreifen und sagen: […] So ist halt der Umgang hier und das hat mit deiner Person nichts zu tun.“ (GP3)* | *“It's mostly non-verbal. [...] this interaction and this distance that is maintained [...] because they probably know it differently from their home countries and sometimes they are disappointed [...] and then I have to intervene and say: [...] That's just the way things are here and it has nothing to do with you as a person." (PSW3)* |
| CODE 1C. Cultural mediator role  SUBCODE  C.1. Cultural mediator role in therapy context | *„Das hilft in der Therapiesitzungen zwischen Therapeutin und der Patient auf bessere Basis kommen Konsens kommen um diese Therapie zu erfolgreich fortzuführen.“ (GP7)* | *„Das hilft in den Therapiesitzungen zwischen Therapeutin und Patient auf bessere Basis und besseren Konsens zu kommen um die Therapie erfolgreich fortzuführen.“ (GP7)* | *"This helps the therapist and patient to reach a better basis and consensus in the therapy sessions in order to continue the therapy successfully." (PSW7)* |
| CODE 1C. Cultural mediator role  SUBCODE  C.1. Cultural mediator role in therapy context | *„Ja, aber bei uns leider es ist von in der Schule auch, wo Geschlecht wird nie gesprochen. Alsor wir haben keine wie sagt man, Kunst, nein, wie heißt das, dieses, diese Auferklärung. Jetzt fällt mir nicht der Namen ein. Und von meine Schüler von meine Kinder weiß ich schon, dass die Auferklärung bekommen. Aber bei uns leider fehlte und die Afghanische genauso fehlt das und das ist so eine Bereich, das niemand will darüber reden. Und natürlich, wenn es jetzt offen ist und auf einmal gefragt wird, sie ziehen sich zurück, weil sie wollen darüber nicht reden. Weil sie die Erfahrung haben sie die nicht.“ (GP1)* | *„bei uns wurde leider in der Schule nie über Geschlecht gesprochen. Also wir haben keine Aufklärung. [...] und das ist so ein Bereich, über den niemand reden will. Und wenn es jetzt offen ist und auf einmal gefragt wird, ziehen sie sich zurück, weil sie darüber nicht reden wollen und diese Erfahrung nie gemacht haben.“ (GP1)* | *“Unfortunately, we never talked about gender at school. So we have no sex education [...] and it's an area that nobody wants to talk about. And when it is now open and suddenly asked, they withdraw because they don't want to talk about it and have never had this experience." (PSW1)* |
| CODE 1C. Cultural mediator role  SUBCODE  C.2. Cultural mediator role in bureaucracy context | *„Dadurch, dass die Problem zum Beispiel mit Behörden haben, oder mit dem Sprachen haben, die haben, die wissen viele Sachen nicht, wie in der Schule läuft, wie es mit Ausbildung ist. Wie überhaupt mit Gesundheitssystem ist. Die wussten gar nicht, wie man eine Überweisung holen sollen. Wofür braucht man eine Überweisungsschein? Oder wann bekommt man eine Überweisungsschein? Und auch, dass sie Recht haben so eine Krankenkasse die Karte bekommen.“ (GP1)* | *„Dadurch, dass sie zum Beispiel mit Behörden oder mit der Sprache Probleme haben, wissen sie viele Dinge nicht – Wie es in der Schule läuft, wie es mit einer Ausbildung aussieht, wie es überhaupt mit dem Gesundheitssystem ist. Sie wussten gar nicht, wie man eine Überweisung holen sollte. ‚Wofür braucht man einen Überweisungsschein?‘ oder ‚Wann bekommt man einen Überweisungsschein?‘ und auch, dass sie das Recht haben, eine Krankenkassenkarte zu bekommen.“ (GP1)* | *"Because they have problems, for example, with the authorities or with the language, they don't know many things - how it works in school, how it is with an education, how it is with the health system at all. They didn't even know how to get a referral. 'What do you need a referral slip for?’ or ‘When do you get a referral slip?” and also that they have the right to get a health insurance card." (PSW1)* |
| CODE 1C. Cultural mediator role  SUBCODE  C.2. Cultural mediator role in bureaucracy context | *„zeitaufwendig. Muss man wirklich Zeit lassen“ (GP1)* | *„zeitaufwendig […]“ (GP1)* | *„Time consuming […]“ (PSW1)* |
| CODE 1C. Cultural mediator role  SUBCODE  C.2. Cultural mediator role in bureaucracy context | *„Und wir brauchen im Moment auch jemanden, der sie zu gedulden finden.“ (GP4)* | *„jemand, der ihnen Geduld vermittelt“ (GP4)* | *"someone who teaches them patience" (PSW4)* |
| CODE 1D. Motivation  SUBCODE  D.1. Facilitating hope and/or promoting independence | *„Ja und versuchen bis dem Therapeuten zu Ende ist, sie selber die Patienten selbstständiger zu machen, und sagen ok, das machst du selber. Du schaffst das.“ (GP8)* | *„Ja und natürlich versuche ich, dass die Patient:innen selbstständiger werden. Ich sage, ok, das machst du selber. Du schaffst das.“ (GP8)* | *“Yes, and of course I try to make the patients more independent. I say, okay, you do it yourself. You can do it.“ (PSW8)* |
| CODE 1D. Motivation  SUBCODE  D.1. Facilitating hope and/or promoting independence | *„Ja, vielmals, also, wenn ich mich denke, viele sind depressiv, die sind hoffnungslos und genau, sie wissen nicht, was in der Zukunft gestaltet wird. Ob die Zeiten vorbei geht oder nicht. Und viele Sachen von meinen Erfahrungen erzähle ich in Deutschland, wie bei mir war.“ (GP1)* | *„Wenn ich an mich [zurück] denke, viele [Geflüchtete/Neuangekommene] sind depressiv, sind hoffnungslos und wissen nicht, wie sich die Zukunft gestaltet. Ob diese Zeit jemals vorbei geht oder nicht. Und viele von meinen Erfahrungen und wie es bei mir war in Deutschland [anzukommen], erzähle ich ihnen.“ (GP1)* | *"When I think [back] to myself, many [refugees/new arrivals] are depressed, hopeless and don't know what the future holds. Whether this time will ever be over or not. And I tell them many of my experiences and how it was for me [to arrive] in Germany." (PSW1)* |
| CODE 1D. Motivation  SUBCODE  D.2. Motivation for therapy | *„ja, sie kommen erste, zwei, drei Termine vielleicht. Hmmm... sie brauchen nicht so viel motivieren, aber danach sie brauchen, weil sie dachten das ist die Ergebnis, kommt so schnell. Also wenn nach drei, zwei Termine sie fühlen, das ist noch keine große Entwicklung, und dann sie brauchen noch mehr motivieren. Das braucht lange, oder sowas. Oder manche Patienten habe keine Lust, keine Lust einfach zu ein Termin kommen, oder haben viel zu tun. Ich muss immer motivieren, und erklären, dass es immer helft, aber braucht viel Geduld.“ (GP2)* | *“[...] sie kommen [zu den] ersten zwei, drei Terminen vielleicht. […] sie brauchen nicht so viel Motivation [von außen], aber danach brauchen sie das, weil sie dachten, dass das Ergebnis sehr schnell kommt. Also wenn sie nach drei, zwei Terminen fühlen, dass noch keine große Entwicklung da ist, brauchen sie noch mehr Motivation. ‚Das braucht lange‘, oder sowas. Oder manche Patienten haben keine Lust zu einem Termin kommen, oder haben viel zu tun. Ich muss immer motivieren und erklären, dass es immer hilft, aber viel Geduld braucht.“ (GP2)* | *“ [...] they come [to the] first two or three appointments maybe. [...] they don't need so much motivation [from outside], but after that they need it because they thought the result would come very quickly. So if after three or two appointments they feel that there hasn't been much development yet, they need even more motivation. 'It takes a long time', or something like that. Or some patients don't feel like coming to an appointment or have a lot to do. I always have to motivate them and explain that it always helps, but that it takes a lot of patience." (PSW2)* |
| CODE  1E. Emotional support | *„wenn die merken, dass ich oft erreichbar, oder die Nachrichten per WhatsApp lese. Habe ich gemerkt, ja, sie, sie fühlen sich nicht alleine.“ (GP6)* | *„wenn die merken, dass ich oft erreichbar bin oder die Nachrichten per WhatsApp lese habe ich gemerkt: ja, sie, sie fühlen sich nicht [mehr so] alleine.“ (GP6)* | *"When they realized that I was often available or read the messages […], I noticed: yes, they don't feel [so] alone [anymore]." (PSW6)* |
| CODE  2.A. Negative Experience (NE)  SUBCODE  A1. NE and “factor PSW” | *„ ‚Du, das mach ich jetzt nicht‘ oder ‚Das ist nicht meine Aufgabe‘. Und wenn man dann aufgelegt hat, hat man immer gedacht: ‚Ok, jetzt hab ich ihm oder ihr das Herz gebrochen, vielleicht hätte ich doch hier da etwas machen können‘." (GP3)* | *„ ‚Du, das mach ich jetzt nicht‘ oder ‘das ist nicht meine Aufgabe‘. Und wenn man dann aufgelegt hat, hat man immer gedacht: ‚Ok, jetzt habe ich ihm oder ihr das Herz gebrochen, vielleicht hätte ich doch hier oder da etwas machen können.‘ “ (GP3)* | *" ‘You know, I'm not going to do that now‘, or ‘That's not my job. ‘ And when you hung up, you always thought, ‘Okay, now I've broken his or her heart, maybe I could have done something here or there.‘ " (PSW3)* |
| CODE  2.A. Negative Experience (NE)  SUBCODE  A1. NE and “factor PSW” | *„oder sie sagen oft, da brauche ich zum Beispiel mehr Hilfe. Das ist einfach für mich ist schwierig, da wo die was brauchen, zur finanziellen Hilfe. Da kann ich leider nicht ihnen helfen und dann bin ich der Meinung, dass hier ein bisschen enttäuscht von mir, weißt du was ich meine, so ist es.“ (GP8)* | *„[…] sie sagen da brauche ich zum Beispiel mehr Hilfe. Das ist für mich schwierig, da sie oft finanzielle Hilfe brauchen. Da kann ich ihnen leider nicht helfen und dann bin ich der Meinung, dass sie ein bisschen enttäuscht von mir sind […].“ (GP8)* | *"[…] they say I need more help, for example. That's difficult for me because they often need financial help. Unfortunately, I can't help them and then I think they are a bit disappointed in me [...]." (PSW8)* |
| CODE  2.A. Negative Experience (NE)  SUBCODE  A1. NE and “factor PSW” | *„Ich muss ehrlich sein, manchmal ich denke immer nach, wie macht der, er ist noch traurig, oder vergleiche ich immer meine persönliche Geschichte mit die Patientensgeschichte, so weiter. Aber habe ich eine Übung für mich selber gemacht, hm ja, manchmal wenn ich schaffe, sitze ich alleine kurz vor, nach dem Therapie, ich gehe nicht zurück nach Hause und sitze ich alleine und ich versuche alles, ja. Manchmal weine ich, manchmal ja, gucke ich an die Bilder: Die erste Bilder aus meinen Heimat oder Eltern oder so.“ (GP6)* | *„[…] manchmal vergleiche ich meine persönliche Geschichte mit der Geschichte des Patienten […]. Manchmal mache ich eine Übung für mich, nach der Therapie bevor ich nach Hause gehe. Manchmal weine ich und manchmal schaue ich Bilder aus meiner Heimat oder Eltern […] an.“ (GP6)* | *"[...] sometimes I compare my personal story with the patient's story [...]. Sometimes I do an exercise for myself after therapy before I go home. Sometimes I cry and sometimes I look at pictures of my home or parents [...]." (PSW6)* |
| CODE  2.A. Negative Experience (NE)  SUBCODE  A2. NE and “factor patient” | *„[…] wenn sie von der Armee, oder Soldaten, oder Verhaftung erzählen, über den die Sachen, ja, für mich ist es schwierig“ (GP6)* | *„[…] wenn sie von der Armee, Soldaten oder von Verhaftung erzählen, - diese Sachen sind für mich schwierig“ (GP6)* | *"[…] when they tell about the army, soldiers or arrest, - these things are difficult for me" (PSW6)* |
| CODE  2.A. Negative Experience (NE)  SUBCODE  NE and “factor patient” | *„Und erstens ist es traurig, dass dabei anzusehen, dass die Menschen am Boden sind. Und zweitens ein Hand zu strecken und sie dann zum Aufstehen bitten, ist auch nicht ganz einfach, weil da kommt nur Ablehnung und da kommt ein Energie, oder ein Gefühl: ‚Lass mich doch in Ruhe, Ich glaub das dir jetzt nicht, du bist ja auch nur wegen deinen Gunsten hier‘.“ (GP3)* | *„Erstens ist es traurig anzusehen, dass die Menschen am Boden sind. Eine Hand ausstrecken und sie zum Aufstehen bitten ist auch nicht ganz einfach. Weil da kommt nur Ablehnung und da kommt eine Energie, oder ein Gefühl: ‚Lass mich doch in Ruhe, Ich glaub das dir jetzt nicht, du bist ja auch nur zu deinen eigenen Gunsten hier‘.“ (GP3)* | *"First, it's sad to see people down. To hold out a hand and ask them to get up is also not very easy. Because there comes only rejection and there comes an energy, or a feeling: 'Leave me alone, I don't believe you now, you're only here for your own benefit'." (PSW3)* |
| CODE  2.B. Positive Experience (PE)  SUBCODE  B1. PE and “factor PSW” | *„fachlich was lernen, sprachlich was lernen. Der Umgang mit schwierige Situationen was lernen. Psychologische Sachen lernen, es ist so, sowas von lehrreich für mich.“ (GP3)* | *„fachlich was lernen, sprachlich was lernen. Den Umgang mit schwierigen Situationen lernen. Psychologische Sachen lernen, es ist sowas von lehrreich für mich.“ (GP3)* | *"Learn something professionally, learn something language wise. Learning how to deal with difficult situations. Learning psychological things, it's so instructive for me." (PSW3)* |
| CODE  2.B. Positive Experience (PE)  SUBCODE  B1. PE and “factor PSW” | *"Das ist für mich persönlich sehr schön, weil ich generell auch im Leben so ne Rolle sehr gern auch nehme. Auch meine Familie gegenüber, auch meinen Kollegen gegenüber. Ich möchte das einfach, dass alle nach vorne gucken und sich nach vorne auch bewegen." (GP3)* | *"Das ist für mich persönlich sehr schön, weil ich generell auch im Leben so eine Rolle sehr gern einnehme. Auch meiner Familie und meinen Kollegen gegenüber. Ich möchte das einfach, dass alle nach vorne gucken und sich nach vorne bewegen." (GP3)* | *"This is very nice for me personally, because I generally like to take on such a role in life. Also towards my family and my colleagues. I just want everyone to look ahead and move forward." (PSW3)* |
| CODE  2.B. Positive Experience (PE)  SUBCODE  B1. PE and “factor PSW” | *„Also für mich war sehr gut, sehr gute Erfahrung. Jetzt hab ich so viel Jahre hier und merk ich schon, wer was benötigt oder wie weit darf man reingehen oder der Abstand kann man besser schätzen. Und man, wie sagt man, wie nah man ihn ran zukommen. Ich merke jetzt besser, als am Anfang und die Rolle was wir haben oder ich habe, kann ich besser, als am Anfang. Kann ich besser zurecht kommen, als am Anfang.“ (GP1)* | *„Also für mich war [es] sehr gut, [eine] sehr gute Erfahrung. Jetzt bin ich so viele Jahre hier und merke schon, wer was benötigt oder wie weit ich reingehen darf, den Abstand kann man besser einschätzen. [...] Ich merke das jetzt besser […] und [mit] der Rolle, […] die ich habe, komme ich besser zurecht als am Anfang.“ (GP1)* | *"So for me [it] was very good, a very good experience. I've been here for so many years now and I already realize who needs what or how far I'm allowed to go in, I can judge the distance better [...] I realize this better now than at the beginning and [with] the role I have [...] I get along better than at the beginning." (PSW1)* |
| CODE  2.B. Positive Experience (PE)  SUBCODE  B1. PE and “factor PSW” | *„[…] weil, ich weiß, dass ich der Person oder die Person helfen kann, und das ist für mich unbezahlbar“ (GP8)* | *„[…] weil, ich weiß, dass ich der Person helfen kann, und das ist für mich unbezahlbar“ (GP8)* | *"[…] because, I know that I can help the person, and that is priceless to me" (PSW8)* |
| CODE  2.B. Positive Experience (PE)  SUBCODE  B1. PE and “work with the coordination office” | *„Also, diese, ja, diese Schulungen sind enorm wichtig.“ (GP1)* | *„Ja, diese Schulungen sind enorm wichtig.“* (GP1) | *“These training courses are extremely important.” (PSW1*) |
| CODE  2.B. Positive Experience (PE)  SUBCODE  B1. PE and “work with the coordination office” | *„Also das war sehr sehr hilfreiche Schulung. Ja das ist da wird man also das Gefühl du bist nicht also in kaltes Wasser reingeschmissen sondern du bist richtig also du hast eine Blick also du wirst nicht komplett sagen wir also aber du wirst mindestens die Basis haben also wie kommst du wie Kommunikation was ist deine wie kannst du dich schützen also diese persönliche Schützen sie achten also hier sie achten schon drauf in der Projekt […]“ (GP5)* | *„Das waren sehr sehr hilfreiche Schulungen. Ja das ist das Gefühl, dass du nicht ins kalte Wasser reingeschmissen wirst […]." (GP5)* | “*[…] very, very helpful training courses. Yes, that's the feeling that you're not thrown in at the deep end* *[…].” (PSW5)* |
| CODE  2.B. Positive Experience (PE)  SUBCODE  B1. PE and “work with the coordination office” | *"Supervision ist absolut am schönsten, ist als Übersetzung oder als Gesundheitspaten. Das ist wirklich für mich, ich liebe das. Ich werde immer Zeit nehmen, wenn es möglich ist […]."(GP8)* | *"Supervision ist absolut am schönsten, ob als Übersetzerin oder als Gesundheitspate. […] ich liebe das. Ich werde mir immer Zeit dafür nehmen, wenn es möglich ist. […]." (GP8)* | "*Supervision is absolutely the best, whether as a translator or as a PSW. […] I love it. I will always participate when I can […]."* (PSW8) |
| CODE  2.B. Positive Experience (PE)  SUBCODE  B1. PE and “work with the coordination office” | *"Gut. Kommunikativ, hilfreich, weil, diejenige, die weniger Erfahrung haben durch die andere […] kriegen sie Rat oder Tipp, wie sie mit der Situation umgehen können."(GP7)* | *"Gut. Kommunikativ, hilfreich. Weil diejenigen, die weniger Erfahrung haben durch die anderen […] Rat oder Tipps (bekommen), wie sie mit der Situation umgehen können." (GP7)* | "*Good. Communicative, helpful. Because those who have less experience […] get advice or tips from others on how to deal with the situation.” (*PSW7) |

**Quotes used as anchor examples in the codes set: Interviews Peer Support Workers (PSW)**

Below is a list of all the quotations used as anchor examples in the code system, translated from the original wording into Standard German and from here into English.

| **Code** | **Original** | **Standard German** | **English** |
| --- | --- | --- | --- |
| CODE  Task areas | *„Oder ich bin also natürlich verantwortlich für die ganzen Termine, die ich hier oder bei den Ärzten vereinbare. Da wo ich wirklich einfach immer den Patienten sage ein paar Tage vorher, hey wir haben da einen Termin. Bitte denke dran. Komm doch pünktlich oder soll ich dich abholen.“ (GP8)* | *„Ich bin verantwortlich für alle Termine, die ich hier und bei den Ärzten vereinbare. Ich sage den Patienten dann immer ein paar Tage vorher, dass wir da einen Termin haben und er daran denken soll. Komm doch pünktlich oder soll ich dich abholen.“ (GP8)* | *“I am responsible for all the appointments I schedule here and sith the doctors. I always tell the patients a few days in advance that we have an appointment and he should remember that. He should be punctual or I offer to pick him up.” (PSW8)* |
| CODE  Positive Experience (PE) | *„Ja, belastend ist für mich immer, die Menschen, die heftig leiden, egal unter was. Wenn ich Menschen seh, die kaputt sind, früh morgens, wenn man sieht, wie sie da zusammengeschrumpft sind, oder weinen oder nicht gepflegt aussehen und so. Das zieht mich runter und das belastet mich sehr, ja. Nur das. (GP3)* | *„Belastend ist für mich immer das heftige Leid der Menschen, egal unter was. Wenn ich Menschen sehe, die früh morgens kaputt sind, wenn man sieht, wie sie da zusammengesunken sind, oder weinen oder nicht gepflegt aussehen und so. Das zieht mich runter und belastet mich sehr. Nur das.“ (GP3)* | *“What is always burdensome for me is the instense suffering of people, no matter the cause. When I see people who are worn out early in the morning, when you see them slumped over, crying, or looking unkempt and so on. That brings me down and burdens me a lot. That’s all.” (PSW3)* |
| CODE  Negative  Experience (PE) | *„Aber jetzt ich sage ne, das Leben ist wirklich schön. Man muss nur ein bisschen mehr dran arbeiten und versuchen den anderen zu helfen. Es gibt sehr viele Punkte, wo ich sage, dass hat mir wirklich sehr glücklich gemacht. Als ich ja, hier, in dem Projekt, arbeite, ja, so.“ (GP8)* | *„Aber jetzt sage ich, das Leben ist wirklich schön. Man muss nur ein bisschen mehr daran arbeiten und versuchen den anderen zu helfen. Es gibt sehr viele Punkte, bei denen ich sage, dass es mich wirklich sehr glücklich gemacht hat. Als ich hier in dem Projekt arbeite.“ (GP8)* | *“But now I say, life is really beautiful. You just have to work at it a little harder and try to help others. There are many points where I say it really made me very happy. When I was working here on the project.” (PSW8)* |
| CODE  Factor PSW | *„Aber jetzt gerade, ich kann mir sehr gut vorstellen, dass ich nicht in meiner Vergangenheit reinrutsche.“ (GP8)* | *„Aber jetzt gerade kann ich mir sehr gut vorstellen, dass ich nicht in meine Vergangenheit zurückfalle.“ (GP8)* | *„But right now, I can very well imagine that I won‘t fall back into my past.” (PSW8)* |
| CODE  Factor patient | *„Es hängt auch von jedem Patienten an und welche Fall ist. Aber momentan ist es toll eine junge Iraner, der auch sehr stark traumatisiert war, schlechte Erlebnis gehabt hat und war auch in Psychiatrie hier und aber langsam bei ihm geht zu Verbesserung seine Gesundheitszustand. Und er besucht Deutschkurs.“ (GP1)* | *„Es hängt auch von jedem Patienten ab und was es für ein Fall ist. Aber momentan ist es toll. Der Patient ist ein junger Iraner, der auch sehr stark traumatisiert war, schlechte Erlebnisse gehabt hat und hier auch in der Psychiatrie war. Aber langsam verbessert sich sein Gesundheitszustand und er besucht einen Deutschkurs.“ (GP1)* | *“It also depends on each patient and what kind of case it is. But right now it is great. The patient is a young Iranian, who was also severely traumatized, had bad experiences, and was also in psychiatry here. But slowly, his health condition is improving, and he is attending a German course.” (PSW1)* |
| CODE Working with coordination office | *„War ganz gut, ich hab da überhaupt keine Probleme. Wenn ich irgendwas, wenn ich irgendwas brauche, oder wenn ich merke ok, das wird für mich sehr viel. Dann nehme ich Kontakt auf, und dann das klappt alles…“ (GP3)* | *„War ganz gut. Ich habe da überhaupt keine Probleme. Wenn ich irgendetwas brauche, oder wenn ich merke, dass es mir zu viel wird, dann nehme ich Kontakt auf und dann klappt das alles.“ (GP3)* | *“It was quite good. I don’t have any problems at all. If I need anything, or if I realize that it is getting too much for me, I get in touch and then everything works out.” (PSW3)* |
| CODE Working with healthcare system | *„Diejenigen, die verstanden haben was ein Gesundheitspate, haben sie gut gefunden….“ (GP7)* | *„Diejenigen, die verstanden haben was ein Gesundheitspate ist, fanden ihn gut.“ (GP7)* | *“Those who understood what a PSW was found it beneficial.” (PSW7)* |
| CODE  value for patients | *„Was ich erreicht habe jetzt Nachhinein wenn ich dann denke... Bessere qualitatives Leben. Der Patient vor der Therapie oder vor meiner Arbeit war ausgeliefert ausgesetzt von Gefahren und keine Ahnung. Drohungen, gesundheitliche Gefahren. Und durch diese Arbeit und Tätigkeit und dann Therapie sie haben dann profitiert dass sie dass sie vielleicht 100prozentig geheilt sind und dadurch besser besseres Leben haben qualitatives Leben oder erleichtertes Leben so. Das ist meine Meinung.” (GP7)* | *„Im Nachhinein betrachtet, habe ein besseres qualitatives Leben erreicht. Der Patient war vor der Therapie oder vor meiner Arbeit allgemeinen und gesundheitlichen Gefahren und Drohungen ausgesetzt. Durch diese Arbeit und Tätigkeit und Therapie haben sie dann profitiert, sodass sie vielleicht 100 Prozent geheilt sind und dadurch ein besseres, qualitatives oder erleichtertes Leben haben. Das ist meine Meinung“ (GP7)* | *“In retrospect, I have achieved a better quality of life. Before the therapy or before my work, the patient was exposed to general and health risks and threats. Through this work, activity, and therapy, they benefited, perhaps resulting in them being 100 percent healed and thus having a better, qualitative, or relieved life. That is my opinion.” (PSW7)* |
| CODE  Coping strategies | *„Ich versuche daraus irgendwas zu lernen. Ich versuche das Positive zu gucken und ich versuche das irgendwie mit meiner Hilfe kompensieren. Und für sie auch schöner machen und das macht es für mich auch schöner. Meistens muss ich auch mir sagen: "ja diese Person hat leider Pech gehabt". Weil es sind auch Sache passiert, manchmal, die nicht rückgängig werden“ (GP3)*  *„da muss man einfach nur sagen: "Ja, es ist traurig und du musst einfach dankbar sein, dass dir sowas nicht passiert ist". Und einfach hoffen, dass diese Person auch drüber hinwegkommt, irgendwie.“ (GP3)* | *„Ich versuche daraus irgendetwas zu lernen. Ich versuche das Positive zu betrachte und das irgendwie mit meiner Hilfe zu kompensieren. Und für sie (die Patienten) es schöner zu machen, das macht es für mich auch schöner. Meistens muss ich mir auch sagen, dass die Person leider Pech gehabt hat. Es sind nämlich manchmal auch Sachen passiert, die nicht rückgängig gemacht werden können.“*  *„Da muss man sich einfach nur sagen: Ja, das ist traurig und du musst einfach dankbar sein, dass dir sowas nicht passiert ist. Und einfach hoffen, dass diese Person auch irgendwie darüber hinwegkommt.“ (GP3)* | *“I try to learn something from it. I try to look on the positive side and somehow compensate for it with my help. And to make it nicer for them (the patients), that makes it nicer for me too. Most of the time I also have to tell myself that the person has unfortunately had bad luck. Sometimes things happen that cannot be undone.” (PSW3)*  *“You just have to tell yourself: yes, that’s sad and you just have to be grateful that it didn’t happen to yourself. And just hope that this person will somehow get over it.” (PSW3)* |
| CODE  Future | *„Was ich mir wünsche? also ich wünsche mir sehr, dass die Supervision mindestens einmal pro Monat stattfindet, wenn das möglich ist, sollen alle kommen…“ (GP8)* | *„Was ich mir wünsche? Also ich wünsche mir sehr, dass die Supervision mindestens einmal pro Monat stattfindet. Wenn das möglich ist, sollen alle kommen…“ (GP8)* | *“What do I wish for? Well, I really wish supervision takes place at least once a month. If possible, everyone should attend…” (PSW8)* |
